# Boosting plant immunity with CRISPR/Cas

**DOI:** 10.1186/s13059-015-0829-4

**Published:** 2015-11-19

**Authors:** Angela Chaparro-Garcia, Sophien Kamoun, Vladimir Nekrasov

**Affiliations:** The Sainsbury Laboratory, Norwich Research Park, Norwich, NR4 7UH UK

## Abstract

CRISPR/Cas has recently been transferred to plants to make them resistant to geminiviruses, a damaging family of DNA viruses. We discuss the potential and the limitations of this method.

See related Research: http://www.genomebiology.com/2015/16/1/238

*Geminiviridae* are a family of DNA viruses that infect a diversity of plants. These insect-transmitted viruses can cause destructive diseases in crop plants and have been described as a curse to food security. Until now, limited progress has been made with developing crop varieties resistant to geminiviruses. In the current issue of *Genome Biology*, Ali et al. [1] report on a new strategy towards improving plant resistance to geminiviruses using the bacterial CRISPR/Cas system.

## Problematic development of geminivirus-resistant crops

Geminiviruses are single-stranded DNA (ssDNA) viruses with genomes of about 3 kb that carry few transcription units and rely on the host machinery to function [2]. Once inside the plant cell, the virus starts its cycle of DNA replication and accumulation followed by virus assembly and movement [2]. Strategies to control geminiviruses include chemicals to limit insect vector populations, RNA interference, expression of mutated or truncated viral proteins, expression of peptide aptamers that bind viral proteins, and conventional breeding of resistant crop cultivars [3–5].

Given the high prevalence of mixed viral infections, engineering broad-spectrum resistance must target common steps along the viral cycle. One such step is replication. During this process, viral ssDNA is released into the nucleus and converted to a double-stranded DNA (dsDNA) intermediate that undergoes rolling-circle DNA replication initiated by the viral replication initiation protein (Rep) [2]. One approach for broad-spectrum geminivirus resistance is expression of mutated or amino-terminally truncated Rep protein, which confers some level of resistance by repression of the viral Rep promoter or by exerting a dominant-negative effect on the formation of complexes with the wild-type Rep protein or the replication enhancer protein (REn; also known as C3) [3]. Another strategy is suppression of viral gene expression by activating host RNA interference mechanisms. Here, a virus-derived hairpin dsRNA targeting homologous viral sequences is expressed in the plant cell and processed into small interfering RNAs that subsequently guide the host silencing machinery to the viral genome [3]. Also, artificial zinc finger nucleases have been successfully developed to target and cleave a conserved sequence motif in geminiviruses so as to inhibit replication of several viruses in the model plant *Nicotiana benthamiana* [6].

Conventional plant breeding has been partially successful in delivering resistant varieties to geminiviruses. However, conventional breeding is time and labor intensive and can be complicated by the fact that resistance traits are often controlled by multiple genetic loci [5]. In addition, both conventional breeding and transgenic strategies face the problem of resistance durability brought about by the capacity of geminiviruses to evolve quickly.

Ali et al. [1] describe a fundamentally different approach to engineering resistance against geminiviruses in plants. This and two other recent publications [7, 8] report on converting the bacterial CRISPR/Cas immune system into a tool for this purpose.

## CRISPR/Cas confers resistance to geminiviruses in plants

The CRISPR/Cas system originates from prokaryotic organisms and acts as an adaptive immune system to protect them against invading foreign DNA, such as phages, by cleaving the nucleic acid by an RNA-guided DNA nuclease in a sequence-specific manner [9]. Recently, the CRISPR/Cas system has become a tool of choice for genome editing applications in various organisms, including plants [10].

**Fig. 1:**
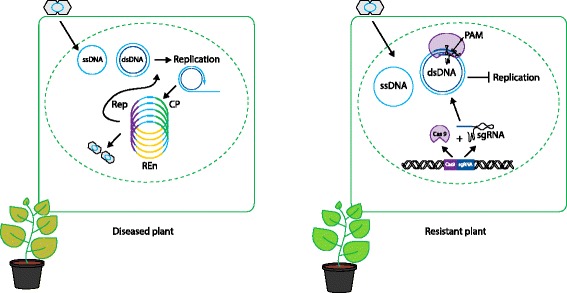
Schematic representation of geminivirus replication in a diseased and a CRISPR/Cas engineered resistant plant. In the plant cell of a diseased plant (*left*), infection geminivirus particles (virions, *gray double hexagons*) release the viral single-stranded DNA (*ssDNA*) into the plant nucleus (*green dashed ellipse*). Host DNA polymerase mediates the synthesis of the complementary strand, resulting in viral double-stranded DNA (*dsDNA*) molecules. Transcription of dsDNA leads to production of Rep protein, which initiates viral replication via rolling-circle replication (*purple circle surrounded by blue open circle*). Multiple cycles of viral replication (*colored circles*) generate new ssDNA that can re-enter replication or can be packaged into virions. In contrast, plant cells expressing a CRISPR/Cas construct that carries sgRNAs targeting sites in the viral genome (*right*) will become resistant to virus infection. The Cas9–sgRNA complex will target the viral dsDNA for cleavage, inhibiting viral replication. *Cas* CRISPR-associated, *CRISPR* clustered regularly interspaced short palindromic repeats, *dsDNA* double-stranded DNA, *sgRNA* single guide RNA, *ssDNA* single-stranded DNA

Ali et al. [1], Ji et al. [7], and Baltes et al. [8] have demonstrated portability of the CRISPR/Cas system to plants to confer enhanced resistance to geminiviruses. Table 1 and Fig. 1 summarize the three studies.

**Table 1:** Comparative summary of the three papers on CRISPR/Cas-induced resistance to geminivuruses in plants

	Ali et al. [1]	Baltes et al. [8]	Ji et al. [7]
Geminivirus species	TYLCV, BCTV, MeMV	BeYDV	BSCTV
Geminivirus delivery	Transient (*Nb*)	Transient (*Nb*)/stable transgenics (*Nb*)	Transient (*Nb*)/stable transgenics (*Nb*, *At*)
sgRNA expressed from:	TRV	T-DNA	T-DNA
Cas9 expressed from:	T-DNA	T-DNA	T-DNA
Geminivirus virulence assay	Semi-qPCR, RCA, Southern blot	GFP, CFUs (*Escherichia coli*)	qPCR, Southern blot
Geminivirus virulence test in systemic tissue	Yes	Yes	Yes
Geminivirus symptoms assayed	Yes, in transient	Yes, in stable transgenic lines	Yes, in stable transgenic lines

*At Arabidopsis thaliana*, *BCTV* beet curly top virus, *BeYDV* bean yellow dwarf virus, *BSCTV* beet severe curly top virus, *CFUs*, colony-forming units, *GFP* green fluorescent protein, *MeMV* Merremia mosaic virus, *Nb Nicotiana benthamiana*, *qPCR* quantitative PCR, *RCA* rolling-circle amplification, *T-DNA* transfer DNA, *TRV* tobacco rattle virus, *TYLCV* tomato yellow leaf curl virus

Cas9 nuclease and single guide RNA (sgRNA), which is an engineered fusion of the dual RNA that directs Cas9 to its DNA target, were expressed in planta. Ali et al. [1] engineered sgRNAs targeting open reading frames encoding the viral Rep and coat CP proteins as well as the conserved non-coding intergenic region (IR), which carries a hairpin structure and serves as the origin of replication. sgRNAs targeting the IR region proved to be the most efficient in bringing down the titer of tomato yellow leaf curl virus (TYLCV). The other two publications [7, 8] reported variable success with targeting different sequences in geminiviral genomes. Importantly, a cumulative reducing effect on the viral copy number was achieved by co-expressing two sgRNAs in the plant [1, 8]. Interestingly, Ali et al. [1] were able to target three viruses at the same time, TYLCV, beet curly top virus (BCTV), and Merremia mosaic virus (MeMV), using an sgRNA matching an invariant sequence within the IR region.

Dampening of viral copy number and symptoms by CRISPR/Cas can be explained by several mechanisms, which are not self-exclusive: a) Cas9/sgRNA binds to an important viral genetic element, such as origin of replication, and thus blocks access of replication proteins to this element; b) Cas9/sgRNA cuts the viral dsDNA and thus interferes with its replication; or c) Cas9/sgRNA mutagenizes the viral genome through the error-prone non-homologous end joining (NHEJ) DNA repair pathway that is recruited by the cleaved viral DNA.

All three studies described mutations, mostly small deletions, in the viral DNA sequences targeted by CRISPR/Cas. It was necessary to demonstrate that the viral mutations detected upon CRISPR/Cas expression were introduced into the freely replicating virus rather than the transgenic input T-DNA delivered by the transformation agent *Agrobacterium tumefaciens*. To address this issue, Ali et al. [1] used TYLCV virion preparations to inoculate CRISPR/Cas-expressing plants. They demonstrated that CRISPR/Cas triggers mutations and interferes with the copy number of freely replicating virus. Nevertheless, field trials remain necessary to determine whether CRISPR/Cas can make plants more resistant to geminiviruses in the natural environment.

## Limitations of using CRISPR/Cas for resistance against geminiviruses

There are several advantages of using CRISPR/Cas to confer geminivirus immunity in plants. CRISPR/Cas allows simultaneous targeting of a single or multiple genetic loci in one or several geminiviruses [1, 7, 8]. The simplicity and robustness of the CRISPR/Cas technology will make it possible to respond to newly emerging strains by deploying appropriate sgRNA transgenes into a crop. However, despite clear advantages, the use of the CRISPR/Cas technology for engineering geminivirus-resistant crops is associated with significant challenges. First, transgenic crops expressing CRISPR/Cas may not be favorably perceived by regulators resulting in high commercialization costs. As a consequence, the strategy of using CRISPR/Cas for resistance to geminiviruses may be commercially viable for major field crops, such as maize, but not cost-effective for crops grown on a smaller scale, such as tomato. Second, constitutive expression of Cas9 and sgRNA(s) may result in off-target mutations in the crop genome that may build up over time. Also, the strategy of using several sgRNAs to target multiple viruses may further increase the rate of off-target mutations, and the guide sequence within the sgRNA transgene may mutate so additional off-target mutations may get introduced. Overall, we need to better understand the rate and degree to which CRISPR/Cas off-target mutations arise in plants.

An intriguing question is whether expressing CRISPR/Cas in crops will exert an enormous selection pressure on geminiviruses and, as a result, accelerate their evolution. The CRISPR/Cas system may select for synonymous or neutral nonsynonymous mutations in targeted coding sequences that would enable the virus to escape cleavage. It is also possible that CRISPR/Cas-resistant mutations arise within targeted conserved noncoding sequences, such as the invariant sequence in the IR, for example when compensatory mutations arise in the Rep protein. It should be pointed out that the CRISPR/Cas system is mutagenic by nature and thus acceleration of virus evolution can be expected. The CRISPR/Cas system may also enhance recombination between distinct geminiviruses when plants get infected by multiple virus strains. Such recombinogenic effect is another risk factor that needs to be taken into account.

## Concluding remarks

The Ali et al. [1], Ji et al. [7], and Baltes et al. [8] papers further illustrate the versatility of sequence-specific nucleases in plant biotechnology and the creative potential unleashed by the routine implementation of CRISPR/Cas in plant biology. As always, benefits and risks need to be carefully evaluated. The new method needs to be considered in the context of other alternatives for managing geminivirus diseases, especially in the developing world. As the pressure to feed a growing world population intensifies, we may have to resort to all the tools at our disposal.

## Abbreviations

Cas: CRISPR-associated
CRISPR: clustered regularly interspaced short palindromic repeats
dsDNA: double-stranded DNA
IR: intergenic region
Rep: replication initiation protein
sgRNA: single guide RNA
ssDNA: single-stranded DNA
TYLCV: tomato yellow leaf curl virus
